# What do adults with visual impairment mean by well-being? Identifying the building blocks of well-being in the context of visual impairment

**DOI:** 10.3389/fpsyg.2024.1395636

**Published:** 2024-08-22

**Authors:** Nikki Heinze, Ffion Davies, Sarah York, Stephanie Hoi-Ying Chan, Derek Farrell, Renata S. M. Gomes

**Affiliations:** ^1^BRAVO VICTOR, London, United Kingdom; ^2^Blind Veterans UK, London, United Kingdom; ^3^Northern Hub for Veterans and Military Families Research, Department of Nursing, Midwifery and Health, Faculty of Health and Life Sciences, Northumbria University, Newcastle, United Kingdom

**Keywords:** well-being, wellbeing, model, sight loss, visual impairment

## Abstract

**Background:**

Well-being has become a key outcome of health and support services for adults with visual impairment (V.I.). However, there is a lack of consensus on how well-being is conceptualized and assessed in V.I. research, if it is defined at all. A shared understanding of what well-being means in the context of adult V.I. is essential to enable comparison of findings across studies and collaboration between support organizations.

**Methods:**

This article reports findings from a series of four online focus groups with adults with V.I. and one with practitioners working in the field of adult V.I. The focus groups explored what participants meant by well-being and which factors impacted their well-being. A total of 17 adults with V.I. and five practitioners took part. A list of all components of well-being, factors that impact well-being, and items in the protective buffer that may mitigate the impact of factors on well-being were extracted from the data.

**Results:**

Despite the noted difficulty in defining well-being and disagreement around the extent to which well-being was universal or individual, a preliminary model of well-being emerged from the focus group discussions. The core of well-being reflects an overall feeling of contentment arising from a positive evaluation of how one is feeling, how one is feeling within oneself, and how one is feeling about one’s life. Factors relating to balance/equilibrium, health, mood, other people, the self, and a sense of security and purpose can positively or negatively impact well-being. This impact may be mitigated by a protective buffer consisting of one’s mood, mindset, ability to cope, resilience, and acceptance. Many items were discussed in multiple roles, e.g., as a component of well-being or factor.

**Conclusion:**

This research took a bottom-up approach to explore what well-being means in the context of adult V.I. The role of certain items and the structure of the proposed model of well-being will need to be confirmed in future research with stakeholders across the V.I. sector.

## 1 Introduction

In the United Kingdom (UK), the number of people living with visual impairment (V.I.) is projected to reach approximately 4 million by 2050 (Pezzullo et al., 2018). V.I. has been associated with poorer quality of life (QoL), sleep, mental health, and mental well-being (Tabandeh et al., 1998; Fenwick et al., 2012; Kempen et al., 2012; Mcmanus and Lord, 2012; Zhang et al., 2013; Van Der Aa et al., 2015; Schliermann et al., 2017; Frank et al., 2019).

Well-being is a crucial outcome of health and social services (The Scottish Government, 2015). The UK’s National Health Service (NHS) adopted a framework focusing on health and well-being not only as part of their service delivery but also for their employees (NHS England, 2021). The “5 steps to well-being” model, adopted and disseminated by the NHS to improve patient well-being, suggests connecting with other people, staying physically active, learning new skills, giving to others, and paying attention to the present moment (Aked and Thompson, 2011; NHS, 2022). Several UK sight-loss charities offer well-being support in addition to vision rehabilitation. For example, the Royal National Institute of Blind People (RNIB) runs a Helpline and Sight Loss Advice Service offering Mental Wellbeing Check-ups (RNIB, n.d.), and Blind Veterans UK (BVUK) runs Wellbeing Centers for their membership (BVUK, 2024). A review found a high prevalence of remotely delivered well-being-focused services offered by sight-loss charities during the COVID-19 pandemic, including welfare, counseling and befriending calls, peer support, and physical exercise (Jones et al., 2022).

Historically, models of well-being have focused on how people feel (hedonia) and how they function (eudaimonia): subjective well-being (SWB; Diener, 1984; Diener and Suh, 1997), for instance, consists of affective (pleasant/unpleasant affect) and cognitive (life satisfaction) components, while psychological well-being (PWB; Ryff, 1989) and self-determination theory (SDT; Ryan and Deci, 2001), two eudaimonic models, consist of autonomy, environmental mastery, positive relationships with others, purpose in life, realization of potential and self-acceptance, and autonomy, competence, and relatedness, respectively. More recent models have combined hedonia and eudaimonia to reflect a more holistic picture of human well-being. For instance, the PERMA model consists of hedonic (Positive emotions) as well as eudaimonic elements (Engagement, Relationships, Meaning, and Accomplishments) (Seligman, 2012), while flourishing comprises emotional, psychological, and social well-being (Keyes, 2002). Others have moved away from hedonia and eudaimonia to conceptualize well-being as the balance point between the challenges and the resources available to an individual (Dodge et al., 2012) or have used related concepts such as happiness (Linton et al., 2016), health (Linton et al., 2016), QoL (Lent, 2004; Cooke et al., 2016; Linton et al., 2016), and wellness (Cooke et al., 2016; Linton et al., 2016).

These models do not consider the potential impact of V.I. Yet V.I. is associated with a range of challenges that may not be captured by well-being models and tools that have been developed with and for the general population. For instance, acquired sight loss requires individuals to adjust to their new circumstances. This includes recreating their identity, relearning and adapting everyday activities and hobbies, as well as roles within their family, employment, and social groups. The process of adjusting to and accepting acquired sight loss has been compared to grief (Bergeron and Wanet-Defalque, 2013). High levels of depression have been found during this period (Senra et al., 2015), while acceptance of one’s V.I. has been associated with lower levels of depression and better well-being (Bergeron and Wanet-Defalque, 2013). Beyond the adjustment period, negative public attitudes pose a significant barrier to participation in everyday life for people with V.I. (Fisher et al., 2018). Indeed, there is evidence of poorer psychological well-being when assessed with vision-specific rather than general measures because respondents may feel worried about future vision loss (vision-specific) but not about the future in general (Pinquart and Pfeiffer, 2011).

However, a scoping review showed a lack of consensus in the way well-being has been defined and assessed in V.I. research (Heinze et al., 2022). This lack of consensus has practical implications. For research, it impacts the comparability of findings across studies, design of interventions to improve well-being, and evaluation of the effectiveness of interventions. For practice, it impacts on cross-organizational collaborations in the provision of support for adults with V.I. This becomes apparent when considering the example of an individual who is referred from organization A, which uses a depression scale to assess well-being, to organization B, whose interventions are designed around a conceptualization of well-being as anxiety, to receive well-being support. A shared understanding of well-being endorsed by adults with V.I. and practitioners working with them is essential for innovation and collaboration. The current article reports findings from a series of focus groups which explored how adults with V.I. and V.I. practitioners define well-being. Their role in supporting adults with V.I. gives practitioners useful insights into the challenges and support needs associated with V.I. A small sample of practitioners was therefore included in this research to gain a different perspective on well-being in the context of V.I. In addition, several structural considerations guided the exploration of well-being: conceptualizing a single indicator may be too narrow to gain a comprehensive insight into an individual’s well-being. A further consideration relates to the distinction between states or indicators of well-being, termed ‘components’ in this article, and determinants or ‘factors’ that impact well-being (Linton et al., 2016). For instance, Lambert et al.’s (2019) multi-dimensional measure of national well-being includes an assessment of physical activity and contact with nature/green spaces, which arguably constitute factors that impact well-being rather than well-being itself. While factors may be usefully included in well-being assessments to guide possible interventions, a careful distinction must be made between what is a component of well-being and what is a factor to avoid further blurring the understanding of well-being.

## 2 Materials and methods

Ethical approval for this research was granted by the Faculty Research Ethics Committee at Northumbria University (approval date 05/02/2023, project number 2619). There is no definitive answer as to the ideal number of focus groups (FGs) in qualitative research, although research suggests that three to six FGs are sufficient to discover 90% of all themes (Guest et al., 2016), while saturation can be reached from four to eight FGs (Guest et al., 2016; Hennink and Kaiser, 2022). Moreover, this research forms part of a wider research program that aims to (1) agree on a definition of well-being in the context of V.I. and (2) identify or develop a tool to assess well-being based on this definition. As part of this program, a scoping review which explored how well-being has been operationalized in V.I. research (Heinze et al., 2022), and the current study were conducted as preliminary data-gathering exercises in preparation for a Delphi study with adults with V.I. and practitioners aimed at agreeing on a definition of well-being.

Qualitative data were collected in four FGs with adults with V.I. and one FG with practitioners between 9 May 2023 and 15 June 2023. All FGs took place online using Microsoft Teams to reduce the need for travel. Each FG lasted approximately 1.5 h and consisted of two moderators (NH, FD) and a maximum of five participants to maximize the time each participant had to voice their opinion. FD has extensive experience moderating group conversations on Teams as a community team leader for BVUK.

### 2.1 Materials

A topic guide was designed to explore participants’ understanding of well-being (submitted as Supplementary material). Broad questions explored what participants understood well-being to mean, including whether they thought that well-being differed or was the same for people with and without V.I., and which factors impacted their well-being positively and negatively.

### 2.2 Participants

A convenience sample was recruited through the researchers’ professional networks, social media, and contacts in the UK V.I. sector. Participants were eligible to take part if they were aged 18+ (i), had a V.I. (defined as reduced vision which cannot be corrected by glasses/contact lenses), or were a practitioner with at least 2 years’ experience of working with adults with V.I. (e.g., as an ECLO, ROVI, ophthalmologist, optometrist, charity support worker, etc.) (ii), agreed to the FG being audio-recorded (iii), and were able to provide informed consent (iv).

### 2.3 Procedure

Potential participants were sent an electronic or audio copy of the participant information sheet and invited to an initial phone call with the lead researcher (NH) to go over questions about the research, confirm their eligibility, discuss their accessibility preferences, and explain the rights of research participants and the importance of confidentiality. Participants were advised that they may know other participants in the group through the various V.I. charities who helped with recruitment. Participants who agreed to participate were then asked to complete a consent and demographics questionnaire online (hosted on SmartSurvey) or over the phone with the lead researcher. To enable data linking, the questionnaire asked for participants’ names; however, these were replaced with the participants’ IDs once the data had been downloaded from the survey platform. Participants could join the FG by clicking on a link sent to their email or by being dialed into the call using their preferred telephone number. Teams displays the names of registered users; thus, instructions were sent to participants to join as guests or provide telephone numbers to be dialed into the FG if they did not wish for their names to be visible to other participants. Cameras were disabled for all participants, except the two moderators, to avoid video-recording participants and to ensure equal access for participants with varying degrees of V.I. At the start of the FG, participants were reminded about confidentiality and invited to agree on a set of ground rules to ensure a positive experience for all participants. At the end of the FG, participants were reminded about data confidentiality and the list of support organizations in the participant information sheet if they were affected by the discussions.

### 2.4 Data analysis

Teams provides a transcript of audio-recordings. During transcript checking, names and identifying information were removed or replaced with participant IDs. Data analysis aimed to extract a comprehensive but manageable list of components and factors which could be further tested in a subsequent Delphi study. While the data analysis was not informed by a specific theoretical framework, thematic analysis as a method was adapted for the data analysis (Braun and Clarke, 2006; Clarke and Braun, 2017). After an initial phase of familiarization with the data in the transcripts, verbatim descriptions of well-being and factors were extracted by two researchers (NH and SC). Codes were derived by grouping together and assigning a label to similar quotes from each FG. For instance, references to feeling upset, down, or gutted were labeled as negative emotions, while feeling okay, happy, or relaxed were labeled as positive emotions. The initial lists of codes were compared, and discrepancies were reviewed in the transcript. The labels and associated quotes were reviewed in several rounds and grouped under overarching categories. For instance, positive and negative emotions both relate to mood and, as such, were grouped under the category of mood. A notable difficulty is related to the complexity of determining the role of certain items. The FGs explored components and factors of well-being. However, the descriptions of well-being and its factors offered by some participants suggested that there may be an alternative role for some items, which appeared to mitigate the impact of factors on well-being. As such, these items appeared to act as a protective buffer. There is evidence of third variables being implicated in the relationship between predictor variables and mental health, either as mediators or moderators. For instance, mastery and self-esteem have been found to mediate the impact of V.I. on depression (Maaswinkel et al., 2020), while negative affect has been found to moderate the impact of stress and positive affect on depression (Nima et al., 2013). The difficulty for participants and coders then lay in distinguishing between components, factors, and items which mitigate the impact of factors, with some (e.g., mood) being mentioned as all three. Member checking was used to ensure the accuracy of the codes extracted for each FG. Items and domains were further reviewed when combining the lists for each FG.

## 3 Results

### 3.1 Sample characteristics

A total of 17 adults with V.I. and five practitioners took part in the FGs. The mean age of participants was 56.1 (*SD* = 12.4) years, ranging from 24 to 75 years. Two participants preferred to give their age as a range (both selected 40–50 years old). Most participants were male (54.5%), white British (81.8%), educated to undergraduate degree level (36.4%), in paid employment (40.9%), and married/in a civil partnership (54.5%). Most were registered as severely sight impaired or blind (94.4%) and had developed V.I. after the age of 18 (50.0%). The most common cause of V.I. were conditions relating to retinal dystrophy such as retinitis pigmentosa or Leber congenital amaurosis (22.2%, *n* = 4). Six participants (27.3%) reported no comorbid physical health conditions, and 17 (77.3%) reported no mental health conditions. High blood pressure (36.4%, *n* = 8), chronic pain (31.8%, *n* = 7), and diabetes (27.3%, *n* = 6) were the most common physical health conditions, and two participants (9.1%) reported having depression. Table 1 provides an overview of the demographic characteristics of participants by subgroup. There were no statistically significant differences between the two groups except for age (*p* = 0.019), which reflects the working age of practitioners.

**Table 1:** Participant characteristics by subgroup.

	Adults with V.I.	Practitioners
*n*	17	5
Age^1^	*U* = 11.5, *p* = 0.019	
*M (SD)*	59.1 (12.2)	47.0 (8.6)
Range	24–75	35–57
Gender	Fisher’s exact *p* = 0.699	
Female	35.3% (6)	60.0% (3)
Male	58.8% (10)	40.0% (2)
N/A	5.9% (1)	—
Ethnicity	Fisher’s exact *p* = 0.210	
White British	88.2% (15)	60.0% (3)
Pakistani	5.9% (1)	—
Indian	5.9% (1)	—
White Scottish	—	20.0% (1)
Prefer not to say	—	20.0% (1)
Education	*U* = 52.5, *p* = 0.446	
No formal qualifications	5.9% (1)	—
GCSE/O-Level	5.9% (1)	—
A-level/advanced highers	1.8% (2)	—
Apprenticeship, vocational qualification, NVQ, or HND	23.5% (4)	20.0% (1)
Undergraduate degree	29.4% (5)	60.0% (3)
Masters, PhD	23.5% (4)	20.0% (1)
Employment status	Fisher’s exact *p* = 0.102	
In paid employment	23.5% (4)	100.0% (5)
Self-employed	5.9% (1)	—
Unemployed	11.8% (2)	—
Retired	47.1% (8)	—
Long-term sick or disabled	5.9% (1)	—
Doing voluntary work	5.9% (1)	—
Marital status	Fisher’s exact *p* = 0.724	
Married or in a civil partnership	47.1% (8)	80.0% (4)
Divorced	29.4% (5)	20.0% (1)
Never married or registered a civil partnership	17.6% (3)	—
Prefer not to say	5.9% (1)	—
Relationship status^2^	*n* = 9	*n* = 1
Living with a partner	11.1% (1)	—
In a relationship but not living with a partner	11.1% (1)	100.0% (1)
Not currently in a relationship	66.7% (6)	—
Prefer not to say	11.1% (1)	—
Registration status^2^		*n* = 1
Registered severely sight impaired/blind	94.1% (16)	100.0% (1)
Registered sight impaired/partially sighted	5.9% (1)	—
V.I. onset^2^		*n* = 1
Born with V.I.	29.4% (5)	100.0% (1)
Before the age of 4	5.9% (1)	—
Between the ages of 4 and 12	5.9% (1)	—
Between the ages of 12 and 18	—	—
After the age of 18	52.9% (9)	—
Do not know	5.9% (1)	—

^1^Two participants preferred to give their age as an age range (40–50). ^2^Comparative analysis was not conducted due to small subsample size in the practitioner group (*n* = 1).

### 3.2 Nature of well-being

The concept of well-being was not universally accepted by all participants.

*…the term well-being is not in my lexicon. It just is not there because when I hear the term well-being, I visualize people wanting me to build the Taj Mahal out of matchsticks and grow a tomato plant or something like that, which I do not want to do. **WB25, FG04***

In addition, participants across the groups acknowledged the difficulty and lack of consensus in how well-being has been defined.

*[what is well-being] I have asked many people this question over the last couple of weeks, and every single person has given a different answer. For some people it’s health. Other people, it’s wealth. For other people, it's family. Could be financial problems. **WB26, FG03***

*I can’t find a decent definition. I went for a well-being talk with a lady and she said there are Five Pillars of Well-Being. And I did the ultimate research: I asked Google, and I got a different five pillars. Then I asked the smart speaker and that gave me another three different definitions of well-being. **WB25, FG04***

*I think, it's difficult to define. And I agree with the fact that it is a very individual thing, and everybody is responsible for their own well-being. **WB15, FG01***

This may be partially due to the structural complexity of well-being and the reciprocal relationship between some components and factors. For instance, a participant in FG02 defined well-being as “*feeling positive about life and having the energy to do the things that I want each day*” and went on to describe how feeling positive and its opposite, feeling down, can impact on what they saw as factors, which can in turn contribute to well-being:

*I think it's having a healthy mind. Feeling positive, feeling confident to tackle life and to do all the things that I want […] There might be a day when I'm not feeling physically strong, but I have got the energy. But I can kind of still feel well, and I can function. But if I feel mentally that I'm not where I want to be, then that can also affect my physical well-being. Because if I'm feeling down, if I'm not feeling confident, if I'm not positive, if I'm emotionally upset, then that also impacts on my ability to kind of get up and go. And I don't wanna exercise. I’m just kind of fixed in one place and I just don't have the energy to want to do anything. So, for me—and they do go hand-in-hand. But mentally for me, for my well-being, I feel that does have quite an impact. **WB10, FG02***

There was a tendency for participants to focus on activities when discussing components and factors of well-being, which is perhaps reflective of the challenges in defining well-being.

*I just think of well-being as mind and body and trying to look after them both. And say, for example, with your body to eat the right things and make sure that you get plenty of rest, exercise and all that sort of thing. And then, from mind wise, make sure that you get plenty of breaks and go for nice walks, go for coffee with friends. **WB15, FG01***

*I would echo what WB15 said. I guess the only thing I'll add is the emotional part of the self-care, for example, journaling and maybe using spirituality, like prayer. Or journaling, which I found quite therapeutic. And of course, the mental side of everything like meeting up with friends, the socializing aspect and physical, like exercising, going out for walks, gardening. **WB04, FG01***

Participants across all groups insisted that well-being was individual and subjective and, as such, could not be defined.

*I still maintain that well-being is something that every individual should define for themselves because it's to do with how they individually feel they are coping with, responding to their own set values and criteria in their life. **WB09, FG01***

Some participants acknowledged that well-being may consist of the same emotions, but how these were experienced may be individual.

*I think everyone has their own sense of well-being. There's not one thing you can put in a pot and say that's your well-being. […] we've all got different ways of being happy or being sad. So, there's not one well-being covers everybody. **WB17, FG03***

Others suggested that it was the factors that impact well-being, which are unique to each individual, but well-being itself was human and therefore universal.

*If I was asked to explain what well-being is, and to me it's an achieved state of positivity. But the causes, the means by which you find that, maybe you stumble across it sometimes, or maybe you generate it by doing some creative writing or whatever it is that you like doing. There are different reasons why it happens or why it's found or different causes. There are also different things that prevent it or reduce it or invert it to negativity, but it is a state of being or in which you are feeling good. **WB01, FG01***

*The actual well-being—contentment, good feeling, happiness, whatever—I think that's probably inside us. A better way of thinking about it is, it’s the same for everybody. It's the external factors and how you deal with them that are different depending on who you are, what you are, physically or mentally. **WB06, FG04***

Some participants raised the temporal nature of well-being, suggesting that well-being fluctuates within the same individual.

*Because when you put the questions to the person, and you ask them to think about how they've been feeling over the last two weeks as well. So, it’s not just literally how’re you feeling in this moment. Because they might have got up, they might have had a really lovely cup of tea, they might be sat in a lovely environment, so, they're feeling better to talk about things. But when you ask them to think about the last two weeks, it will bring to mind that time where they were sat in despair or not feeling good about things, not being able to complete things. **WB04P, FG05***

*I guess it depends on people’s life circumstances as well. Everybody is at a different point and, for example, if somebody’s moving houses, that's quite a big thing or someone job hunting. **WB04, FG01***

Despite these challenges, some participants came to the FGs with fully formed models of well-being, seeing it as the sum of scales relating to physical, mental, and financial well-being, happiness, a sense of control and feeling good about yourself, or as being safe, physically and mentally healthy, and happy.

*I almost see that well-being is a totaliser of the score on a whole bunch of scales. So, you've got the health scale, you've got the mental well-being scale, you've got the financial scale, you've got the how-you-feel-about-yourself scale. And if you took all of those and added the score up, that would be your well-being for that day, for that moment. And if any one of those goes down, then it's a drop in your overall well-being […] But if you were going to write down things that you then ask people on a scale of 1 to 10 today, at this moment, where would you rate your happiness or your financial—are you comfortable financially—and you're trying to measure, which I guess ultimately is the perfect scientist solution to this question of what is well-being, it's a score out of 100 or whatever. But those are the ones I've thought of. Sense of being in control was one, sense of inner happiness, which I think I've talked about, and sense of feeling good about yourself. **WB30, FG03***

*What I mean when I go out and I visit people and talk about their well-being is: are they safe, are they happy, are they well, are they feeling good in themselves, physically and mentally. That they don't have any niggles, that they're not having any major concerns, something's worrying them. **WB04P, FG05***

The following provides an overview of the individual domains and items participants associated with well-being, some of which were discussed as a component, factor, and items mitigating the impact of factors, making their role unclear. Exemplary quotes are included in the narrative and Table 2.

**Table 2:** Components and factors of well-being with supporting quotes.

Item	Role	Quote
Balance/equilibrium	*As a component*	I think as you get older, and as you learn more about life and you get more experiences of what good or bad, or experiences can be, I think it allows you to regulate more how you respond to ups and downs and that keeps the sort of general mean of what people might call well-being a lot more in equilibrium than it might be when you are young or at particularly fraught or euphoric times of your life. **WB09, FG01**
	*As a factor*	When I reflect upon my day, am I satisfied with the way my day went? If I’m not, have I got control over addressing those things, which imbalance my day, made it a rubbish day, or not so good day. And therefore, how can I improve it in the future? But basically, it’s that equilibrium. Am I settled? Am I comfortable with the way my life is? Where it is not, am I in control of changing things to help bring them back to that level of equilibrium where I am comfortable? **WB32, FG04**
Contentment	*As a component*	I think of it as a state of contentment or relaxation or a state of feeling OK. Or an absence, at least, of sort of negativity. But maybe in fact, some kind of positive feeling. **WB01, FG01** The last speaker mentioned the word contentment. I think that’s a very good word to cover well-being. But, again, contentment will be defined individually by different people. I’m actually slightly skeptical of things like happiness and even the reverse of that, the doldrums. I think that what you have got to pitch for is something that is good enough. **WB09, FG01**
Happiness	*As a component*	[Asked what other scales to include in their model of well-being] Happiness and, you know. Happiness, I think, is tied in with all of it. **WB30, FG03** It’s a sense of happiness, a sense of contentment or that kind of all over sense of how you feel at that time. **WB30, FG03**
	*With self and life*	I’ve been away on a driving week last week, doing things I’d never thought was possible since losing my sight. So, my well-being during that week has been pretty high. I’ve been happy. **WB26, FG03** Well, you would feel happier in yourself if your well-being’s good. If your well-being’s not so good, so basically if you are not getting any exercise for the day or something like that, it starts to make you feel down and things like that. **WB24, FG02**
Feeling good	*As a component*	And it’s just a feeling. The end result is a feeling of, feeling good. **WB01, FG01** For me, well-being is having a feeling, feeling positive about life and having the energy to do the things that I want each day. **WB10, FG02**
Mood	*As a component*	[how do you know if your well-being is high/low] It’s realizing the triggers. Some triggers, some things that happen within your life, your daily day that can make your mood drop. Or it can pick your mood up. **WB28, FG03**
	*As affect*	My well-being can very easily be affected by outside factors. Things like negative attitudes can really stress me out. They can really make me feel down sometimes. And that to me is not a good feeling of well-being. **WB07, FG04**
	*As a factor*	You go through, I suppose, different stages of well-being, depending on your moods and achievements or knockbacks. **WB26, FG03** On the other hand, visual impairment certainly does diminish our natural ability or perhaps what we might have done had we not had a visual impairment and that causes a certain amount of frustration, which might actually not improve our well-being, it might actually tip the scale the other way. **WB09, FG01**
	*Going hand-in-hand*	I think it is chicken and egg. And I think if your mood is good then chances are your well-being will be better even though it’s a combination of things. But clearly if, I do not know, a lot of exercise is your thing and that improves your mood, then the whole well-being picture is gonna improve. **WB03, FG03**
	*As a protective buffer*	Depending […] what mood you are in, you deal with those things, the negatives, better or worse. But if you get caught in a bad day and things start going wrong, it can really put you into a tailspin. **WB30, FG03**
Health	*As a component*	But I’d say my well-being was in a good place, partly if I feel good in body and mind as a basis. **WB03, FG03**
	*As body and mind*	Physical well-being as well links in with mental well-being, and mental well-being links in with physical well-being. **WB05P, FG05**
	*As a factor*	For me, physical exercise gives me an element of, whether it’s the endorphins or whatever it is, that really gives a buzz to me, that makes me feel more content with myself than I would be if I wasn’t physically fit. Equally mental health is important. **WB30, FG03**
	*Impact of comorbid conditions*	I do not just have vision impairment. I also have learning difficulties and that was quite profound. **WB04, FG01** But I’m finding that my hearing is a bit of a problem and I’ve been to have it tested. And I’m not quite deaf enough for a hearing aid. I find that far more frustrating at the moment than I am with my vision. **WB15, FG01** I’m mobility restricted as well, so that does cause a problem which, again, we go back to mental well-being. **WB21, FG02**
Social interaction	*As a factor*	A lot of people I meet are quite often quite socially isolated and that’s usually when people are expressing feelings of unwellness. So, I think that social connectedness is something that can be significant for people. **WB03P, FG05**
Communication	*As a factor*	You can be in a room full of people and hear them talking and the noise, but you could be the loneliest person within the room. **WB28, FG03**
Support	*As a factor*	I would say talking to someone who you trust and someone that is willing to listen is a good sounding board for, to help you come back from having that low. **WB28, FG03** My dog is my rock, comfort blanket. Again, she knows everything, and she picks up on how I’m feeling. If she feels that I’m a little low, she’ll come and snuggle into you. And stroking her, it eases the tensions or whatever. **WB26, FG03**
Sense of belonging/connectedness	*As a factor*	But I just want a full life, a purpose of life. Because if I feel I’m contributing and belong to the community, then that is going to be very positive for my well-being. **WB12, FG02** [what is well-being] I guess, from myself, connectedness. I think it was WB03P, saying that people being able to be connected and accepted within a group as an equal and for that to be enabled. I think for people with a V.I. that’s not always the case. **WB01P, FG05**
Attitudes, behaviors	*Impact on well-being*	I do think it’s important that we balance the negative stories about other people because there are also fantastic other people out there. An example: my girlfriend and myself were down, went to Morrisons, got ourselves there. I’d only headbutted one lamp post on the way, we got to the car park, got a bit lost in the car park and this guy came over and he said ‘oh, you want to get to the entrance?’ So, he guided us to there and he said, ‘do you need a hand to do your shopping?’ He helped us do all our shopping, he then said, ‘how are you getting home?’ We said we’ll get a taxi, ‘No, I’ll give you a run home’. He did all of that of his own back, just because he was a nice guy. […]. **WB30, FG03** Negative attitudes from other people, whether it’s lack of services, whether it’s. All of these things definitely have that grinding negative effect on my mental health and if I did not have the impairment, yes, it still would in different ways, but I do not think it would have half of it. I would not have half of them if I had sight. **WB10, FG02**
	*Examples of negative attitudes and behaviors*	Because we acquired a disability, often people will assume that we cannot do things, or I have a couple of people who are frightened to come to my house. At least they assume that there’s gonna be a problem and I could not possibly make them a cup of tea, or I could not possibly cook them lunch. **WB12, FG02** People, when you are out and about, and I use a long cane and they just walk straight in front of you or they got the phone up in front of the face, texting, doing whatever. And they walk straight into you and it’s your fault. Those sort of people by the time I get home after being out like that, you are thinking ‘oh, please just let me sit in the dark corner for a while’. But there is—I mean, do not get me wrong, there is good people out there—but there’s also these people who are just, they are insular and they just want to go from A to B or get whatever they need, push you out the way and off they go. ‘he’s blind, he do not care or we do not care about him.’ **WB17, FG03** And what I find is, quite often members of the public will almost impose their understanding of your identity on you, because of societal stereotypes. **WB03P, FG05**
	*Lack of education/confidence*	[friends/family] I think they do struggle with it. They do not know how to treat you really, do they? **WB17, FG03** If we get poor treatment in a supermarket or good treatment in a supermarket, it could easily be down to the level of confidence or nervousness that that presumably sighted person is feeling. So sometimes it’s as much about your confidence and well-being coming up against that of the public. So, there’s always a complex picture. **WB03, FG03**
Barriers/opportunities	*As a factor*	For me, fulfillment and opportunity and access to that opportunity, that’s real and they do not feel given to them any more than it is given to anybody else, so it’s not to feel that you are singled out, but actually that’s part of what is should be accepted normally to make sure that people can access opportunities for their well-being. **WB01P, FG05**
Feeling equal and valued	*As a component or factor*	What still happens is, you still do not get listened to […] I would say that is probably the biggest, is other people just not crediting what you say with the same importance or substance that the average person would get. It’s a sort of difference in the credit you receive. **WB03, FG03** They tend to look at disabilities before the look at the people. **WB24, FG02**
Energy/motivation	*As a component*	Also, feeling confident in yourself and feeling like you have the energy to do things. **WB04, FG01** If people find themselves in this space where they almost do not care about anything, which is not to say that they have lost their compass or anything, but it’s just flat. They cannot motivate themselves to do things. I know that is arguably a classic description of depression, and that’s not quite what I mean. **WB03, FG03**
Self-esteem	*As a component*	For me personally, it’s just being comfortable with yourself. **WB26, FG03**
	*As a factor*	And if you lose your job or something, that can just knock things like confidence and self-esteem, because then you think ‘well, how am I going to look after my family and keep I cannot pay my mortgage’ and all that sort of thing. So that has an impact on well-being. **WB15, FG01**
Self-confidence	*As a component*	I think if you have got that ability to feel confident about doing things and if you are able to look forward to things […] I think it’s that combination of contentment, confidence, knowing that they can take control of things. I think those things are signs that a person’s well-being in a good place. **WB03, FG03**
Self-worth	*As a component*	I like the comment about if how you feel makes you feel worthwhile. That sums it up. I quite like that take on it. **WB25, FG04** Because the attitude is, ‘well, you cannot do this, you cannot do that because you need sight’. And they just make you feel worthless. **WB21, FG02** I know when I go to a venue and for some reason I cannot access it, then I get quite irate. It makes me frustrated. So, if that’s a daily, day in-day out, regular occurrence that must impact upon well-being. […] there might be an underlying kind of chipping away at yourself or your self-worth really potentially. **WB01P, FG05** For me, it’s having compassion for yourself and having compassion for other people. Love yourself, because if you cannot love yourself, you cannot love other people regardless of whether they are good or bad or indifferent. **WB28, FG03**
Self-reliance	*As a factor*	I think that sense of having sometimes to depend on others to do the things that you want can be depressing. **WB10, FG02**
Perceived competence	*As a factor*	I find that I get very frustrated [inaudible], is when I’m doing something that I’m perfectly capable of doing, but I. Because I’ve got no sight, I forget I’ve put a glass of wine on the edge of the table or something and you knock it over and. Just those stupid little things. Or you bang your head on the counter when you are bending down to pick something off the floor or. And the number of times I’ve got into a rage with myself, calling myself ‘you silly blind blow’ whatever. **WB30, FG03**
Mindset/attitude	*As protective buffer or factor*	But the real consequence of that is: you brushed it off, another person might walk out that bar and never return and might not even come out of their flat for a week because of a stupid off-the-cuff comment like that. And that would affect their well-being because suddenly they took the chance to go out to a bar, had a stupid comment like that thrown at them and suddenly they are back, weeks trying to build that courage again to walk out their front door. **WB06, FG04**
	*Own response*	So, there’s a situation, it’s how we respond to it. And a bad response—and I do not mind labelling some responses as bad responses—do impact on the person and other people. So, I recognize that responding positively to an adversity, or at least reducing your response so it does not impact on the ones around you, is very important. **WB01, FG01**
	*Examples of positive mindset/attitude*	I also think laughter plays a big part in our well-being, being able to laugh not just at things that might be funny on the television, but at ourselves. Things that we do on a daily basis that maybe we just do not get right the first time and we have cooked a meal and we find maybe something within the meal is not quite cooked the way it should be, but we have eaten it anyway. It’s the ability to be able to laugh at oneself. **WB28, FG03** I think since we are talking about well-being, the trick surely, or the mechanism surely, is always to look for the positive. And when you find a negative [do not] dwell on it. Certainly, make fun of it, write an article about it, that’s great. But after that? If you allow that to be the major thing that comes out of what you are doing, then you, then the well-being is going to drain away and it’s. **WB09, FG01** There are therapies that challenge this, they kind of present other ways of looking at things or alternative thinking styles. And that’s why I realized that I’m thinking some of this for this reason, but actually it is possible to look at it in a different way, maybe alleviate it. **WB01, FG01** If something’s not working for you, then [inaudible]. But there’s merit in trying them out. And going for the walk once was good because it [inaudible]. But do not repeat the same trick too often, because it might not work every time. But there are so many other ways of generating well-being to try. **WB01, FG01**
Empowerment	*As a factor*	Frustration is a big ever-present factor, the inability to do things for oneself, feelings of powerlessness, of sorting out quite simple things. **WB01, FG01**
Mastery	*As a component*	But those are the ones [scales] I’ve thought of. Sense of being in control was one, sense of inner happiness, which I think I’ve talked about, and sense of feeling good about yourself. **WB30, FG03**
Acceptance	*As a protective buffer or factor*	I try to be very tolerant of just about everybody, but I accept in life there are some people, who are just bad eggs, but basically—to be polite about them. And I just accept that whereas before I’d get angry with them or about them. **WB32, FG04**
	*Self-acceptance*	I think there’s something about where you are on your sight loss journey and the process of acceptance. I meet a lot of people through my work that are they are unhappy about the fact that they have got acquired sight loss. So, I think that where you are on your journey and where you are, in that process of acceptance makes a difference because if you have got a positive. To me, if you have got a positive outlook and it’s like ‘OK, I’m blind or I’m partially sighted’ and that does make things it can be more difficult to do the things you want to do when you want to do them. But actually, if you are further down the line and you say that, OK, you have to think ahead and you have to plan and that’s just part of what you have to do, that actually you get to the endpoint of where you want to be or whatever you want to do. **WB03P, FG05**
Resilience	*As a protective buffer*	You still have to build yourself a resilient shell. […] I have a set a bit of a resilient shell because I just have to say, ‘you know what? I do not care if I have to ask someone to give me a guide [to the toilet in a work context], that’s just the reality of it. **WB03P, FG05**
Ability to cope	*As a component, protective buffer, or factor*	So, it’s the subjective, it’s the personal, interplay between external forces and our own mental or psychological strength or whatever. Well-being is somewhere around how we how we cope and how we cope is how we feel about ourselves on a longer basis. **WB09, FG01** The problem about that is that well-being, as I understand it, is how we respond to situations, not the situations themselves. And so somehow or other there needs to be one marker for how good or bad the day has been, but the secondary marker about how good or bad has been our ability to cope with that. **WB09, FG01**
Identity	*As a factor*	
Sense of security	*As a factor*	If any bit of that, like WB26 saying, financial or health or your physical or mental well-being, if any bit of that is out of kilter, then it affects your overall sense of well-being. **WB30, FG03**
	*As a component*	[what is well-being] For some people it’s health. Other people, it’s wealth. Other people, it’s family, could be financial problems. **WB26, FG03** What I mean when I go out and I visit people and talk about their well-being is: are they safe, are they happy, are they well, are they feeling good in themselves, physically and mentally. That they do not have any niggles, that they are not having any major concerns, something’s worrying them. **WB04P, FG05**
Sense of purpose	*As a component*	The emotional feelings around life and the feeling purposeful and that life is worth living. **WB07P, FG05**
	*As a factor*	I want to be contributing, so that my life feels it’s got some value, not just for me, but for others around me so that I’m not always receiving. I can give as well. **WB12, FG02** I think, being part of the solutions does kind of—stuff that’s going on in our world, the world, your world—I think can help with that sense of purpose that they are actually useful. I’m not just a passenger. I can be more than that. So, I think they are key elements for people, V.I. or not, I guess. **WB01P, FG05**
	*Derived from caring and helping others*	This is why I do the dementia thing every couple of weeks. It’s because I’m giving to them. I know that they cannot help what’s wrong, and they probably cannot understand it, but I’m there to make sure they are safe, and that makes me feel good. **WB21, FG02** I was running a group this morning for people in dealing with their alcohol recovery because I now help out at a group, helping other people. And I walked away from that this morning feeling it had been a positive. The people in the group had reacted positively and addressed their different issues and I felt I’d helped them. But also, people always say to me ‘oh, thank you for coming to help us’. And I try to say, with no false modesty, it’s helping me as much as it might be helping other people. **WB32, FG04**
Fulfillment	*As a component*	It’s all about what makes us feel good as individuals. And actually, giving us time to feel good, giving us time to be fulfilled. **WB07, FG04**
Meaningful activities	*Examples of meaningful activities*	Since I started volunteering and giving back to others as well as kind of upskilling myself, I’ve felt better within myself. My energy levels have gone up. And whereas I actually thought it would be the opposite, that I would be more tired doing it, but actually, my energy levels have gone up and mentally I feel all of the things that everyone said: just having that sense of purpose and value and yes, stimulus, just something to get up for. **WB10, FG02** For me, music plays a very big part. It can lift your mood. It can also transport you to different times in your life, happy, sad. I love music and I would listen to it to every day, and I find that that helps set me up for the day **WB28, FG03**
	*What is meaningful is highly individual*	I did not do any exercise, hardly any exercise, and I still feel a sense of well-being, even though I do not do any exercise. So, I do not think that is a huge factor. I think you need to be active, but I do not necessarily exercise. **WB10, FG02**
Faith/spirituality	*As a factor*	I guess the only thing I’ll add is the emotional part of self-care, for example, journaling and maybe using spirituality, like prayer. **WB04, FG01**
Enjoyment/fun	*As a factor*	So, I think it’s maintaining that balance of doing things that you enjoy doing whether that is going for a walk, or it might just be sitting in a favorite chair with a good book or something. **WB07, FG04**
Personal goals, expectations, and challenges	*As a factor*	For me, it’s mental stimulus, having a purpose. If I continuously kind of set myself goals in life and have that social interaction and social life, and I’m learning new things, then I feel I could live to 100 because I’ve got the well-being to keep carrying on. But if I did not have that sense of purpose and I did not set myself goals, if I’ve nothing to kind of work toward, I shut down and then I feel mentally depressed and isolated. **WB10, FG02** I also think challenges. Not just everyday challenges, but I mean challenging yourself, like you have with your golf and the other gentleman with his photography. **WB28, FG03**
Achievements	*As a factor*	I’ve recently started playing golf again properly. And hitting a shot right, your head goes ‘Oh brilliant! Well done.’ But then you hit another one, which is rubbish. So, you just go down again. But I find little victories every day make you feel so much better. **WB17, FG03**
	*Through self-praise*	[Asked for more positive factors] I’d say self-praise…. **WB03, FG03** When we talk about the things that show healthy state, I think that ability to sort of congratulate yourself for things that you have done well—we all feel quite silly if we do that—but I think if people are in a place where they can actually say ‘actually, do you know what? It is a really good thing that I’ve made my first bacon sandwich after sight loss for example.’ **WB03, FG03**
Role of V.I.	*Impact on factors*	I think it’s the same with a little extra layer, we are humans first of all. But I think our visual impairment can affect our lives and the things we can do and the attitudes of other people and our own … the effort and hard work that we have to do in order to achieve simple things. **WB12, FG02**

### 3.3 Balance/equilibrium

*Role: Component, factor*

Participants across the FGs referred to balance/equilibrium of factors, between physical and mental health and in life, or even a sense of calm, when asked to give their definitions of well-being.

*But I think it’s also a balance. It’s when things are in proportion: you’ve eaten a meal that’s good and healthy and you’re not on the sugar high. It’s that, almost moderation. You can’t always expect to be exhilaratingly happy, but you don’t want to be [inaudible] stressed all the time either. So, well-being comes in a sort of healthy balance of, everything’s in proportion and you are in good health not—and I don’t mean just physically, I mean all-round. You’re not sleepless, you’re not bad-tempered and worried all the time. **WB12, FG02***

However, views from other participants suggest that balance/equilibrium, for instance, in body and mind, could also be a factor that impacts on well-being.

*Feeling that everything in your world is at one. So, if any bit of that, like WB26 saying financial or health or your physical or mental well-being, if any bit of that is out of kilter, then it affects your overall sense of well-being. I think it’s not one individual item. If you think of like an outer, the outer shell is yourself. And if in the outer shell everything’s calm and in place, then that’s a good state. If everything’s chaotic and mangled and churning around, then that’s perhaps not such a good sense of well-being. **WB30, FG03***

### 3.4 Contentment

*Role: Component*

A repeated component across the FGs was contentment. Some participants defined well-being as an overall state of contentment.

*I got a kind of metaphor in my head of a sunny day that gets little clouds, and the clouds might be big clouds or little clouds, or dark clouds or fluffy clouds. But they’re all the little things that start to creep on your perfect sunny day. And if you’re able to deal with them and cope with them in whatever means you have available, then your well-being, your sense of contentment is greater; if you’re not able to deal with them then it makes life a lot harder. **WB30, FG03***

This overall state may be impacted by the extent to which people feel content with themselves and their life circumstances.

*There’s two elements to well-being, and overwhelmingly, the contentment of dealing with problems that you get with, when you suddenly find [yourself] in bad health, or a breakdown of a relationship, or not achieving the sort of income that you’d set yourself for or the number of friends you [inaudible]. So, it’s how you deal with not achieving that, which add up to good or bad contentment and therefore well-being. **WB09, FG01***

When asked if contentment was a factor or component of well-being, one participant suggested it was both, although their description indicated it may be more relevant as a component.

*I think it’s both. I suppose it definitely impacts on it. For me though, I would say that contentment is also a big part of it, because it’s just means that you’re in a good place with things. It doesn’t mean you have to be sort of bouncing with happiness the whole time, but you’ve got things sorted, you can respond to things, if things are bad, then you can respond in a healthy way to something that’s bad. **WB03, FG03***

#### 3.4.1 Happiness

*Role: Component*

Several participants referred to a sense of happiness when defining well-being.

*It's a whole feeling of being happy. **WB30, FG03***

As with contentment, an overall state of happiness may be impacted by the extent to which people feel happy at that time, with themselves, and with their life.

*But to me, well-being really, I suppose in layman’s terms, would be me being happy and comfy in my life as best as it possibly can with my disability. So, if I’m living happily and comfortably and doing the best I can with what I’ve got. **WB06, FG04***

#### 3.4.2 Feeling good

*Role: Component*

Finally, some participants equated well-being to feeling good, including the extent to which they felt good within themselves and their life.

*[what is well-being?] Feeling good within yourself? **WB24, FG02***

There is overlap between contentment, happiness, and feeling good. Some participants used the terms interchangeably or collectively, whilst others expressed a clear preference.

*The actual well-being—contentment, good feeling, happiness, whatever—I think that’s probably inside us. **WB06, FG04***

### 3.5 Mood

*Role: Component, factor, protective buffer*

Contentment, happiness, and feeling good can reflect mood if they relate to how a person feels at a specific moment. Several participants listed mood when asked what they meant by well-being.

*If I feel like I’ve got a good state of well-being, if I recognize that feeling of positive relaxation, a good mood, I suppose that’s how I see it. That might not be how everyone sees it […] but that’s kind of how I know whether I’m in a kind of contented place or not. **WB01, FG01***

Mood is related to affect. Participants listed a range of positive emotions, such as feeling satisfied, calm, or stress-free, and negative emotions, such as feeling down, anxious, or stressed, when discussing well-being.

*[Well-being] It’s kind of being discussed in a bit more obvious ways. Whether it’s talking to my sister and saying ‘oh, not feeling mentally,’ ‘I'm feeling down’ or ‘I'm anxious’. **WB10, FG02***

*I think well-being is probably the feeling of satisfaction, of calm, of stress free or low stress living, or whatever. I think the things around us impact on it and, as you said, it can be positive, it can be negative. **WB07, FG04***

One participant explicitly defined well-being as the absence of negative affect and the presence of positive affect.

*I think of it as a state of contentment or relaxation or a state of feeling OK. Or an absence, at least, of sort of negativity. But maybe in fact, some kind of positive feeling. **WB01, FG01***

Other participants identified mood as a factor rather than a component of well-being.

*Frustration is a big ever-present factor, the inability to do things for oneself, feelings of powerlessness, of sorting out quite simple things. There’s factors there: frustration in general that could be anything, but particularly around self-reliant, being self-reliant or not as the case may be. [Is frustration a factor or well-being] I think it’s a factor on it. **WB01, FG01***

When asked if mood was a component or factor, other participants felt it went ‘hand-in-hand’ whereby better mood was associated with better well-being.

*It’s hand-in-hand really, if you're feeling low and down, then your well-being is not gonna be at that particular time, is it? **WB26, FG03***

Mood can also be interpreted as mitigating the impact of factors such as negative behaviors.

*One of the good things when I feel good is, I'm more likely to be tolerant of the sort of things that perhaps I’m less tolerant of normally. So, I’ll actually relax on certain areas of preferences or tolerate things I don’t really like. Because I’m feeling in too good a mood to worry or mind. **WB01, FG01***

### 3.6 Health

*Role: Component, factor*

Across the FGs, participants equated well-being to being healthy or feeling good in body and mind.

*[what is well-being] Health. Being healthy. **WB17, FG03***

Participants generally referred to physical and mental health, seeing them as interconnected, although for some, the inclusion of mental health was relatively new.

*[what is well-being] Good mental and physical health. **WB12, FG02***

*And before I used to think of well-being just being physical health and so if I was asked to say, ‘I'm feeling physically OK, I’m feeling well’. But now I know it’s much more holistic than that, that it is very much about mental state for me. **WB10, FG02***

Physical and mental health were also identified as a possible factor impacting well-being.

*[asked for more positive factors] Try and keep yourself as healthy as you can so you can live longer. Keep the biscuit tin higher in the cupboard. **WB28, FG03***

Several participants gave examples of the impact of their V.I. and comorbid physical, mental, and neurodevelopmental health conditions on well-being.

*[does V.I. impact on well-being] Yes, it did. It did at the start because I was really depressed, I was. I thought the world had ended and everything else. **WB24, FG02***

### 3.7 People

*Role: Factor*

A recurring theme across all FGs were other people. Various aspects relating to encounters with other people, including social interaction, support, communication, attitudes, and behaviors, were listed as factors.

#### 3.7.1 Social interaction

Participants across the FGs highlighted the positive impact of social interaction and the detrimental impact of social isolation on well-being.

*I think social interaction and not being isolated and having healthy relationships and friendships with others definitely, for me, it increases my sense of well-being. **WB10, FG02***

#### 3.7.2 Communication

Other participants described how difficulties with communication, particularly eye contact, arising from their V.I. impacted their ability to socialize and form relationships and, thus, their well-being.

*I feel that there is one thing actually that might be particular [to V.I.] and it goes back to what we’ve all said about communicating and whether our words have value and clout, whether we get through to people, how much we have to repeat ourselves, and so on, to be taken seriously. And I think part of that is quite specific to visual impairment just because we don’t have eye contact with people. Literally the brain scientists will tell you how fixed we are on faces and eyes. And how we read people by facial expression and so on and so forth. That’s one of the huge implications of blindness or sight loss, is that all that stuff goes and we’re not even aware how important it is. **WB03, FG03***

#### 3.7.3 Support

Some participants discussed the impact of formal and informal support from their wider community, family and friends, other people with V.I., and even pets, who could act as someone to talk to and possibly ease feelings of social isolation.

*I guess a difference between how well-being is between visually impaired people is the amount of support they get. Some families might see vision impairment differently to others. For example, I’m in the BAME community and my family might see it different to another culture, for example. […] I was abroad from the UK and that's when I didn’t get [educational] support. […] I guess if you live in a city, for example, you might have more support groups for visually impaired people compared to if you live in a town. I think having those support networks is a really important thing as well as. **WB04, FG01***

#### 3.7.4 Sense of belonging/connectedness

Although one participant initially listed connectedness as part of their description of well-being, further contributions suggest that having a sense of belonging or connectedness to a community may be a factor which can impact on well-being.

*He’s always been engaged quite a lot within the [edited] arts community at least, and he’s disengaging a little bit on that. By not having that connectedness, I think, that’s affected his well-being for sure. **WB01P, FG05***

#### 3.7.5 Attitudes and behaviors

A prominent factor across all FGs were the attitudes and behaviors of people. Participants described the positive and negative impact attitudes could have on their well-being.

*My well-being can very easily be affected by outside factors. Things like negative attitudes can really stress me out. They can really make me feel down sometimes. And that to me is not a good feeling of well-being. But if you have a day where things go to plan, and I think again positive attitudes. If you meet someone kind or nice when you’re out, that sort of thing can massively affect how you feel about yourself and the people around you. **WB07, FG04***

Negative attitudes included assumptions about participants’ capability and competence. Others described a lack of social etiquette in their interactions with sighted people, who would walk into them, ask them personal questions, and impose their perceptions onto them.

*It’s almost like the social etiquette between people, that social etiquette either is stretched so it doesn’t exist, so people quite often will ask me really personal questions, or they’ll verbalize their assumptions about how I live my life. And I think, again, for people who become sight impaired, I think that change in public perception is something that you encounter. You encounter it with quite a force. **WB03P, FG05***

Some participants acknowledged that poor behavior may well reflect a lack of education and confidence on the part of the sighted person.

*The thing I mentioned before, coming from a BAME background, I guess it’s the education. That these communities aren’t educated about disability and seeing it differently, in a more positive light. I think those are the two main things that impact my mental health. **WB04, FG01***

#### 3.7.6 Barriers and opportunities

Negative attitudes and behaviors, at a societal level, resulted in barriers and a lack of access to opportunities, which can impact on well-being.

*All these factors we’ve mentioned, the impact on me feeling happy and worthwhile, living my life as happily as I can, as fulfilled as I can, as a totally blind person in a fully sighted world. Because there’s barriers everywhere in all senses of the word to do things. My well-being, if it was to be put in this kind of sense, is to basically, live my life by getting through those barriers in any way which I can to make my life worthwhile, happy and content. **WB06, FG04***

### 3.8 Self

Various items related to self-construal and beliefs. Their role within well-being was unclear; some may be interrelated with other factors.

#### 3.8.1 Feeling equal and valued

*Role: Component, factor*

Negative attitudes, barriers, and a lack of opportunities may impact on the extent to which a person feels equal and valued. Similarly, behaviors such as not giving equal weight to ideas shared by people with V.I. or being ignored in favor of companions may impact the extent to which people feel seen and heard.

*Specially when I go shopping, should it be for groceries or clothes or whatever, I find that the shop assistants will ask my daughter for payment, and it’s me that’s buying it. I feel like I’m invisible sometimes. **WB28, FG03***

But it is not always clear if these are considered factors or components of well-being. For instance, feeling equal and valued may require acceptance from other people. Indeed, one participant suggested that feeling accepted by others may impact feelings of self-worth and well-being.

*It wasn’t great access [to venue], but they made sure it was okay and it was right for us. But that’s not the norm within society, so that must really impact upon your sense of worth within society, your sense of people accepting who you are and accommodating you as another individual. **WB01P, FG05***

#### 3.8.2 Energy/motivation

*Role: Component*

Several participants included having the energy and motivation to do things in their definitions of well-being.

*And for me, well-being is having a feeling, feeling positive about life and having the energy to do the things that I want each day. **WB10, FG02***

#### 3.8.3 Self-esteem

*Role: Component, factor*

Self-esteem reflects how a person thinks others view them and how they value and perceive themselves, based on their opinions and beliefs about their abilities, competence, qualities, physical self, and worth. In other words, self-esteem reflects the extent to which people feel content, happy, good, and comfortable within themselves. For some participants, this was a component of well-being.

*I guess it’s also about self-esteem and what are their self-beliefs. What do I think about myself and what do others think about me? Because that’s quite a big thing. If you think others think negatively about you, that’s gonna impact your mental health. As well looking at yourself in the mirror, for example, body image. That's a big thing as well. [Do you think these are factors or part of well-being?] I think it's part of well-being. **WB04, FG01***

However, other contributions are less clear, suggesting that self-esteem may also be a factor.

*[is self-esteem well-being or a factor?] Well, self-esteem must be a major part of well-being because I take the view that well-being is something that you set for yourself. You mark yourself against your own, against what you think is right for you. But from the conversation we’re having, I believe that well-being should be set as a personal marker. You should be deciding whether you’re doing well or badly day-to-day against your own personal chart and, so self-esteem has a part to play in that, but it should not be a universal mode. It shouldn’t be something that everybody tries to match. **WB09, FG01***

#### 3.8.4 Self-confidence

*Role: Component, factor*

Regarding self-esteem, several participants described well-being as feeling confident in oneself and one’s abilities.

*I think it’s having a healthy mind. Feeling positive, feeling confident to tackle life and to do all the things that I want. **WB10, FG02***

Conversely, other contributions suggested that self-confidence may be a factor that impacts well-being, to the extent that one has the confidence and self-efficacy to deal with difficult situations.

*[people not coming for lunch] And that really effects my well-being because it knocks my confidence and my own self dignity of just being a normal person and having friends to lunch or not having them for lunch. **WB12, FG02***

#### 3.8.5 Self-worth

*Role: Component*

Regarding self-esteem, participants also discussed feeling worthwhile/worthless, valuing and loving themselves, all aspects of self-worth, when describing well-being.

*Worthwhile is a good word. I think if you feel worthwhile, if you feel that what you’ve done in the day is worth something, either to you or to another person, then that is, that kind of sums it up quite well. **WB07, FG04***

#### 3.8.6 Self-reliance

*Role: Factor*

One factor, which may be particular to people with disabilities such as V.I., was the extent to which they felt self-reliant or dependent on others.

*There’s factors there: frustration in general that could be anything, but particularly around self-reliant, being self-reliant or not as the case may be. **WB01, FG01***

#### 3.8.7 Perceived competence

*Role: Factor*

Perceived competence may arise from achieving a task independently or requiring help from others. Feeling capable or incapable may impact on well-being.

*I’ve been away on a driving week last week, doing things I’d never thought was possible since losing my sight. So, my well-being during that week has been pretty high. I’ve been happy. And then I get back to the [edited] and get let loose on the ground floor and I’m pinballing off each wall and getting lost. That’s a bit of a comedown after the highs of doing things that you didn't think you were gonna be doing again. **WB26, FG03***

#### 3.8.8 Mindset

*Role: Factor, protective buffer*

Mindset, or one’s attitude, was a recurring theme across the FGs, but it is not immediately clear from the examples if it is a factor or mitigates the impact of factors.

*The other thing which I think is very important is trying to have a positive mental attitude. […] My partner, she is totally blind as well, but she is fiercely independent, and she really struggles with the idea of asking for help or accepting help. And I’ve been slowly over the last six months, a year encouraging her to say, look, actually if you say yes to getting assistance instead of. It doesn’t mean you’re any less independent. It just means that you’re getting what you want done, done.… if you flip it on its head, you’re also getting an opportunity to meet someone you wouldn’t have met and they’re generally really nice helpful people. And that’s a wee interaction that your day wouldn’t have had, if you hadn’t been blind. So, I try and flip things like that around to have a positive twist to it instead of it being a negative. **WB30, FG03***

Their mindset or attitude could impact on the way participants responded to challenges.

*It did at the start because I was really depressed. I thought the world had ended and everything else. And it wasn’t until I was doing my [inaudible] cane training and realized, OK, I’ve gotta stick. It can tell me if we have to get off the pavement. And I’ve got me ears, I can listen for cars [inaudible]. If I come up against an obstacle, I don’t know how to get around it, I start to go back to my trainer, ask and get told a simple solution for things. The thing is, don’t be afraid to ask. And you’ve got to have a positive attitude to accept blindness. **WB24, FG02***

At least one participant described it as an optimistic (glass half full) vs. pessimistic (glass half empty) outlook on life. Other participants spoke about being able to laugh at oneself and developing a willingness to look for positives, to change how one looks at things, and to try different things.

*For me, it’s, is it a glass half empty or a glass half full? That’s how you can approach your day, whether it’s half empty or half full? **WB28, FG03***

#### 3.8.9 Empowerment

*Role: Factor*

Several participants suggested that the extent to which people with V.I. felt empowered or, on the contrary, powerless, impacted on well-being.

*It means my empowerment is very reduced. My individuality is reduced. I think this has a fundamental bearing on how we feel about ourselves. It’s our ability to operate as a person. The freedom to do that. I think depression is very much caused by loss of empowerment, by loss of ability to put your own points, to be in control of your own destiny and for whatever reason that is diminished will reduce well-being and, ultimately, I fear, lead to lead to depression. **WB09, FG01***

#### 3.8.10 Mastery

*Role: Component, Factor*

Some participants included mastery or a sense of control in their definition or model of well-being.

*It’s that combination of contentment, confidence, knowing that they can take control of things. I think those things are signs that a person’s well-being is in a good place. **WB03, FG03***

However, the extent to which a sense of control has a positive or negative impact on well-being may rely on an awareness of what is within one’s control, making it unclear if a sense of control is a component or factor.

*And if there’s a bit that you’re not content with, is it something you can control? If you can’t control it, then how are you gonna deal with it as it is, rather than get depressed about what you can’t control. It’s learning to accept those bits we can’t control. We cannot control the level of our eyesight, therefore there’s no point in us getting worked up about the fact our eyesight is the state it is. How do we deal with that? **WB32, FG04***

#### 3.8.11 Ability to cope

*Role: Component, factor, protective buffer*

Related to mastery is a person’s ability to cope. Although presented as an integral part of well-being, it is unclear if this participant views a person’s ability to cope with difficulties as a component, factor, or protective buffer.

*I come back to this thing of having fixed points and measuring well-being by how you individually cope with achieving or not achieving those fixed points. So, there’s two elements to well-being, and overwhelmingly, the contentment of dealing with problems that you get with, when you suddenly find [yourself] in bad health or a breakdown of a relationship or not achieving the sort of income that you’d set yourself for or the number of friends you [inaudible]. So, it’s how you deal with not achieving that, which add up to good or bad contentment and therefore well-being. **WB09, FG01***

#### 3.8.12 Acceptance

*Role: Factor, protective buffer*

For things outside one’s control, e.g., other people’s attitudes and behaviors, participants suggested that well-being was derived from acceptance.

*I don’t think I’m quite there with that sort of acceptance and. Sometimes, for instance, the people that talk to you like a 5-year-old when you’re out and about, that really riles me because I. And then I think, ‘well, yes, I should just like WB32, be calm about it, not let them make me angry’. But then the other part of me is saying, ‘why should I be content to accept that people see me as a less person?’ And that they feel they can talk down to me. So, there’s a lot of internal contradictions going on because I think, ‘why should I sit back and accept that people are going to put barriers in my way and talk down to me?’. **WB07, FG04***

The importance of self-acceptance after sight loss was apparent in the participants’ contributions.

*I do miss what I was doing pre-blindness. And when I sort of got home from hospital, I sold all my fishing equipment, my kayak, my computers and consoles. And I wish I hadn’t though. I thought that’s it, life’s over. Sat on my bed for, well, at least six months [inaudible]. Driving is a big one for me. It’s my freedom gone. But I’ve learned that, it’s these things may not be as easy as they were before, but you can still do it. Where there’s a will, there’s a way, isn’t it? **WB26, FG03***

Indeed, acceptance may be an important precursor for well-being, acting as a factor or protective buffer.

*I think people have talked about a process of, there’s a kind of thing of acceptance of the situation and then moving on to a place where you can regain some sense of well-being. And that can be emotional, physical whatever it might be. I think it’s such a complex thing, isn’t it? Someone said it’s so different for everybody, isn’t it? You know, what makes people happy? **WB01P, FG05***

#### 3.8.13 Resilience

*Role: Protective buffer*

Several participants discussed how resilience could mitigate the impact of factors on well-being.

*And I wonder if people’s ability to sort of cope with the factors that will affect their well-being is down to how resilient they are. And some people have a natural resilience that when they meet an obstacle, they can just, ‘right okay, I’ve hit this wall, how do I get round it? How do I get over it? How do I go through it?’ Whereas other people meet a wall and that’s it. That’s the end. That’s the stop. They can’t see round it. How to get round it. How to get through it. And so, I think people’s resilience levels will also have an impact on how well they’re able to cope, their well-being. **WB04P, FG05***

### 3.9 Identity

*Role: Factor*

Participants noted the positive and negative impact of V.I. on identity. While it engendered a sense of identity and belonging, practitioners discussed the challenges associated with having to recreate one’s identity after sight loss.

*Although I agree, talking about protected characteristics, carry a degree of disadvantage but equally they engender some positive things: they also engender identity, membership of an identity and maybe other things. I could say there are all sorts of positive things that have resulted from my having a vision impairment as well and I don’t deny the disadvantage, but I think it’s part of a picture, not the whole picture. **WB01, FG01***

*It is wrapped up in loss of all the different things and, you said it yourself, identity is, you have to reinvent yourself, which is, as an older adult, quite difficult, isn't it? **WB01P, FG05***

Similarly, difficulties associated with one’s roles and responsibilities at any one time, such as providing for a family, were noted as factors that could impact well-being.

*Certainly, as an impact on my well-being in the past was a time when I was running my own business, and for probably 10 years, the business was struggling to be perfectly honest, and I certainly had major concerns about my family and providing for them. During that time, my marriage broke up, the children went to university, and they’re sort of self-supporting. And even though I still had the business problems, they were much less important to me. They did not become the black dog which they had been while I was still fundamentally terrified about providing properly for my family. So, these things do have an interaction. Or there’s a different degree imposed on situations depending on what else is going on in your life. **WB09, FG01***

### 3.10 Sense of security

*Role: Component, factor*

For several participants, well-being was impacted by the extent to which they felt safe because their basic needs were met, or they were financially secure.

*I can’t remember the order of it, the Maslow hierarchy of things, that he in his theory thought that people needed for self-esteem. And I think one of it, is security, isn’t it, and a roof over your head and the fact that you’re looking after your family properly. And that you feel sort of safe and secure. And if you lose your job or something that can just knock things like confidence and self-esteem, because then you think ‘well, how am I going to look after my family and keep I can’t pay my mortgage’ and all that sort of thing. So that has an impact on well-being. **WB15, FG01***

Other participants included a sense of security in their definitions of well-being, e.g., by ensuring their basic needs are met.

*Well-being means that the person has their basic needs met as a fundamental thing, but then that they also feel contented and fulfilled with their life, emotionally and mentally through feeling that their life has purpose and meaning. **WB07P, FG05***

### 3.11 Purpose

#### 3.11.1 Sense of purpose

*Role: Component, factor*

Having a sense of purpose, which allowed participants to feel useful and like they were contributing to society, was a recurring theme across the FGs. For some participants, having that sense of purpose and feeling that their life was worthwhile was a component of well-being.

*I’d probably say if you’re happy and you’re feeling that you’ve done something, you’re worthwhile, then you’re probably doing alright. **WB06, FG04***

Other participants described it as a factor that could impact on their well-being.

*But I just want a full life, a purpose of life. Because if I feel I’m contributing and belong to the community, then that is going to be very positive for my well-being. **WB12, FG02***

Purpose was derived from various sources but was often tied to caring, e.g., for a guide dog, or helping others, including people with dementia, alcohol problems, sighted people, or those with newly acquired V.I., which turned participants’ V.I. into a strength.

*What I particularly love and what really gives me a thrill is when my blindness becomes my strength. When I help newly visually impaired people, people losing their sight. Suddenly what’s been bad is something that I can utilize, that experience of being there myself, and share knowledge and tips. […] In terms of well-being and fulfillment, that is fantastic when I realize I saved somebody the distress or reduced their distress because I’ve been able to give them some help, that gives me a real buzz and sense of well-being if you like. **WB12, FG02***

#### 3.11.2 Fulfillment

*Role: Component, factor*

Having a purpose may give people a sense of fulfillment, which may be a component of well-being.

*Well-being means that the person has their basic needs met as a fundamental thing, but then that they also feel contented and fulfilled with their life, emotionally and mentally through feeling that their life has purpose and meaning. **WB07P, FG05***

However, one practitioner in FG05 described a sense of fulfillment as a factor rather than component.

*People feeling fulfilled and having things that they enjoy doing, that feeds that wellness. **WB03P, FG05***

#### 3.11.3 Hope

*Role: Component*

Purpose and having opportunities may further be tied to a sense of hope, which was identified as a component of well-being in the practitioner group.

*[…] so it’s not to feel that you’re singled out, but actually that’s part of what is should be accepted normally to make sure that people can access opportunities for their well-being. And hope as well, I guess something to look forward to. **WB01P, FG05***

#### 3.11.4 Meaningful activities

*Role: Factor*

In addition to helping others, purpose was derived from a wide range of meaningful activities, such as volunteering or hobbies.

*When you’re considering well-being, should there be some kind of consideration of hobbies or music, or you’re listening to the radio and other things that fill your day, things that give you a sense of purpose? **WB30, FG03***

What was considered meaningful was highly individual.

*I’m not an avid reader at all, so I find it quite hard, but I. One of the things [charity] did teach me was how to use a lathe. And I’m making pins and things like that. So, if I get sort of low, I’ll go and lock myself in my shed and do what I want. If I want to pick up a bit of wood and throw it, I will just make sure I don’t break anything valuable. **WB21, FG02***

Meaningful activities appeared to be related to feeling useful to society, as this example of a painter with recent onset of sight loss shows.

*He’ll draw something and he’ll show me. And he gets quite upset about the fact it’s not as it was. So, that sense of being able to do what he wants to do and, and have something positive in his life has gone, and he’s in a position where he’s finding it very hard to move on from that, into transitioning into a new way of looking at things, and a new way of doing things. And I think his age works against him because he thinks this is just part of the end of his usefulness within society. **WB01P, FG05***

#### 3.11.5 Faith/spirituality

*Role: Factor*

Two participants suggested that their faith or spirituality could impact their well-being because it gave them a sense of acceptance and equality.

*And having a faith and that’s sort of. For me, there’s no. I’m equal in God’s sight with everybody else, however, many disabilities and things I’ve got wrong with me or can’t do or whatever. I’m still regarded as valuable by him. So that gives me some incentive to keep going and hold on tight. **WB12, FG02***

#### 3.11.6 Enjoyment/fun

*Role: Component, factor*

Some participants highlighted the importance of enjoyment and fun in life, although its role in well-being was unclear. Enjoyment could be derived from finding the right meaningful activities and, as such, act as a factor that impacts well-being.

*…my photography swoosh, gone, it’s up there somewhere and I enjoy every minute of photography and it takes me away from stresses and strains of things that might possibly upset me. **WB17, FG03***

In contrast, this participant viewed enjoyment or fun as an important component of well-being.

*[charity] helped me do things and so yes, some of the things we do are just pure fun. But that having fun is that well-being, well, the element of well-being for me…. **WB32, FG04***

#### 3.11.7 Personal goals, expectations, and challenges

*Role: Factor*

Purpose was also related to having goals. These helped participants keep busy and challenge themselves, which could impact their well-being.

*You might set yourself a target for the day or have an expectation for an event or something and it may exceed that, or it may not. And that has a massive influence on your well-being because you have set a bar that may either be exceeded or not reached. **WB09, FG01***

One participant noted the impact of unrealistic goals and expectations on well-being. Therefore, the suggestion was to set goals based on internal rather than external aspirations and ideals.

*I think one of the problems with young people and well-being is that they haven’t absolutely got a clear understanding of what their own markers are, what their own ambitions and everything else are. They set very, very high levels of expectation, which are often acquired from social media and from other sources. And when they don't reach something which is not really very tangible, they begin to be concerned about that and that affects their perception of well-being. **WB09, FG01***

#### 3.11.8 Achievements

*Role: Component, factor*

Having goals was closely related to a sense of achievement. Participants discussed a sense of achievement as a component of well-being, often arising from relearning how to do small things such as cooking food after sight loss, helping others, and meaningful activities.

*So little successes, like your bacon sandwich or you’re not burning yourself when you get your pizza, you still got a ladder, and you still got a means by which you can progress and make yourself have a sense of achievement and a sense of better well-being because you've got your own wee ladder that you've created for yourself. **WB30, FG03***

Other participants described a sense of achievement as a factor impacting well-being.

*What has a positive impact on my day, or on how I feel is achievements. If I find a way of sorting out a computer issue, or I find a way of sorting out a cooking issue by myself, or I find a way of downloading an app on my smartphone and being able to use it without wanting to throw the smartphone through the window, they improve my day. **WB25, FG04***

Although WB25 was not immediately comfortable with the notion of achievements nor well-being but was happy to adopt the term contentment instead.

*I set my day through achievements and if I achieved making myself or batch cooking, I don’t know, chili con carne, I don’t think that’s going to improve my well-being. I just think that’s going to improve my diet. And then if I write an article and I’ll finish that and get that published, that’s fine. And then I do something else, and I sit down and listen to the radio for an hour. It’s achieving something or adapting the way of doing something to achieve something. And if I find a way of boiling rice, so to speak, without scalding myself, that’s an achievement. If I don’t, after three goes, I wait until [edited] my carer turns up, and say, ‘I’ve been trying this, got any clues what I should do?’ And I don't think it's affected my well-being at all. It's just something that I've now got to adapt another way of doing something. Well-being as I said before, doesn't fit, and it doesn't seem to fit what I see in these one-day seminars or courses or talks. Maybe I'm missing something. Maybe I just don't follow phrases like this. **WB25, FG04***

Several participants suggested that an important aspect of cultivating a sense of achievement was self-praise, or the act of acknowledging even little victories after sight loss.

*But it is really important to tell yourself that that's a good thing. And if we can all pick up that habit, however silly it feels, I think on balance things start to get better. But, at the same time that kind of link between your triggers and that ability to congratulate yourself, I think, is quite important. **WB03, FG03***

Again, not all participants felt comfortable with the idea of self-praise.

*I find that self-praise can be a little bit annoying at times. People saying ‘you're doing amazing blah blah blah'. Well, no, not really. I'm doing what I've gotta do just to get through every day there's nothing amazing about it. You've got to do what you've got to do at the end of the day, isn't it? **WB26, FG03***

### 3.12 Role of visual impairment in well-being

As noted by participants, V.I. may impact on the factors of well-being.

*[is well-being different for people with V.I.] For me, the difference might lie in the causes or triggers. The things that give you that feeling of well-being might be derived differently. And equally the things that cause you to feel the opposite of well-being, the negative feelings, they might also be different. Like some of the solutions won't necessarily work the same way for visually impaired people. **WB01, FG01***

For instance, some participants described poor mental health immediately after losing their sight, prompted by the new difficulties associated with carrying out “normal” everyday activities and cherished activities, resulting in a sense of loss and vulnerability.

*When I lost my sight, I lost my job, I couldn't drive, I couldn't play golf, I couldn't do my photography, so you go into a really dark place. Really, really dark. You don't wanna talk to people, you don't wanna do anything. **WB17, FG03***

As part of the process of adapting to sight loss, participants described having to remake their identity and relearn how to do things such as cooking or hobbies.

*One of them, for instance, activity, of course, for us with visual impairments, we're not gonna do all of the same activities, but we adapt to them like we heard WB06 […], but we hear of people doing. They accept the challenge. They're not playing the same tennis that Tim Henman or Andy Murray are playing. They're playing tennis with other people that have adapted to the situation, so I think the principles of the well-being are the same, but it's how we all learn to adapt to them, to implement them for our own well-being, getting back to that. **WB32, FG04***

This suggests that there may be differences in the experiences of people with acquired vs. congenital V.I., although these may not be too big. Indeed, issues relating to communication, barriers, and attitudes affect people with V.I. regardless of onset.

*I guess another difference is the onset of sight loss. I was born with vision impairment, so I haven’t learned how to go about things, I've just done it naturally, cause I've been doing it for 20 plus years. I have friends who recently lost their sight loss and they’re teenagers, and that can be really difficult because they have to learn everything again, they have to learn new things like using a screen reader, using a long white cane, exploring all the options, and getting about. **WB04, FG01***

*I think that there are differences for someone that's never had vision from the people that have experienced a drop, but not as great as you might think, hearing some of these other accounts. **WB03, FG03***

Moreover, V.I. may impact on commonly recommended interventions to improve well-being. For instance, a health-related intervention such as going for a walk may be beneficial for sighted people, but it can have a negative impact on people with V.I. due to associated barriers and a need to adapt certain activities to make them accessible.

*Going for a walk is often recommended as a thing to help you escape from negative feeling and reboot yourself and you can get a lot of positivity out of going for a walk. However, that was something I tried during lockdown as a thing. In fact, where I worked, we were all urged to do it. […] The first time I did it, it gave me lots of ideas and material to write up. So, I came back and wrote a humorous article based on going for a walk around the neighborhood. That worked once. […] Going for walk was annoying every time, was bringing me to a very, very negative place. […] It might have been really good had I been sighted to go for a walk. It might have worked in terms of being a positive experience. However, because of the nature of being a cane user in the way the environment was set up was having the opposite effect, so I had to stop. **WB01, FG01***

## 4 Discussion

This article, summarizing findings from a series of FGs with adults with V.I. and V.I. practitioners, demonstrated the difficulty of discussing and defining well-being. There was some disagreement about the extent to which well-being was universal or individual. A middle ground may be found in a model that ascribes the sensation of well-being as universal but allows for individuality in the extent or strength to which this is experienced and in the factors that impact it at any given moment. None of the participants equated well-being to QoL, and only one equated well-being to wellness/unwellness (“*People feeling fulfilled and having things that they enjoy doing, that feeds that wellness*”—**WB03P, FG05**), both of which tend to be used interchangeably with well-being (Lent, 2004; Cooke et al., 2016; Linton et al., 2016).

There was a focus on activities in many responses when first asked what well-being was. Activities such as going for walks or socializing are arguably not well-being, but they can contribute to well-being by improving physical health and social isolation. This highlights the difficulty in determining the different layers of well-being and at which level different items sat during the coding process. Often, the role of an item, such as health, is not immediately clear. While poor health may impact well-being, the sensation of being healthy or feeling healthy may be part of well-being.

Similarly, striving for and finding a balance in factors may impact well-being, but the feeling of balance is part of well-being. To some extent, the latter reflects existing attempts to conceptualize well-being as the balance point between existing challenges and resources (Dodge et al., 2012). In addition, factors may be interconnected. For instance, participants described the impact of V.I. (health) on their ability to communicate and, thus, have social interactions, while helping others, including sighted people, may increase participants’ feelings of self-worth and competence, both of which are self-related factors. The complexity of distinguishing between components and factors underlines the multi-layered structure of well-being.

### 4.1 Toward a model of well-being in the context of adult V.I.

Although the precise role of many items needs to be confirmed in future research, a preliminary model of well-being emerges from the FGs, consisting of a core of the various components that make up well-being, a protective buffer which mitigates the impact of factors on well-being, and the factors that can impact well-being.

**Figure 1:**
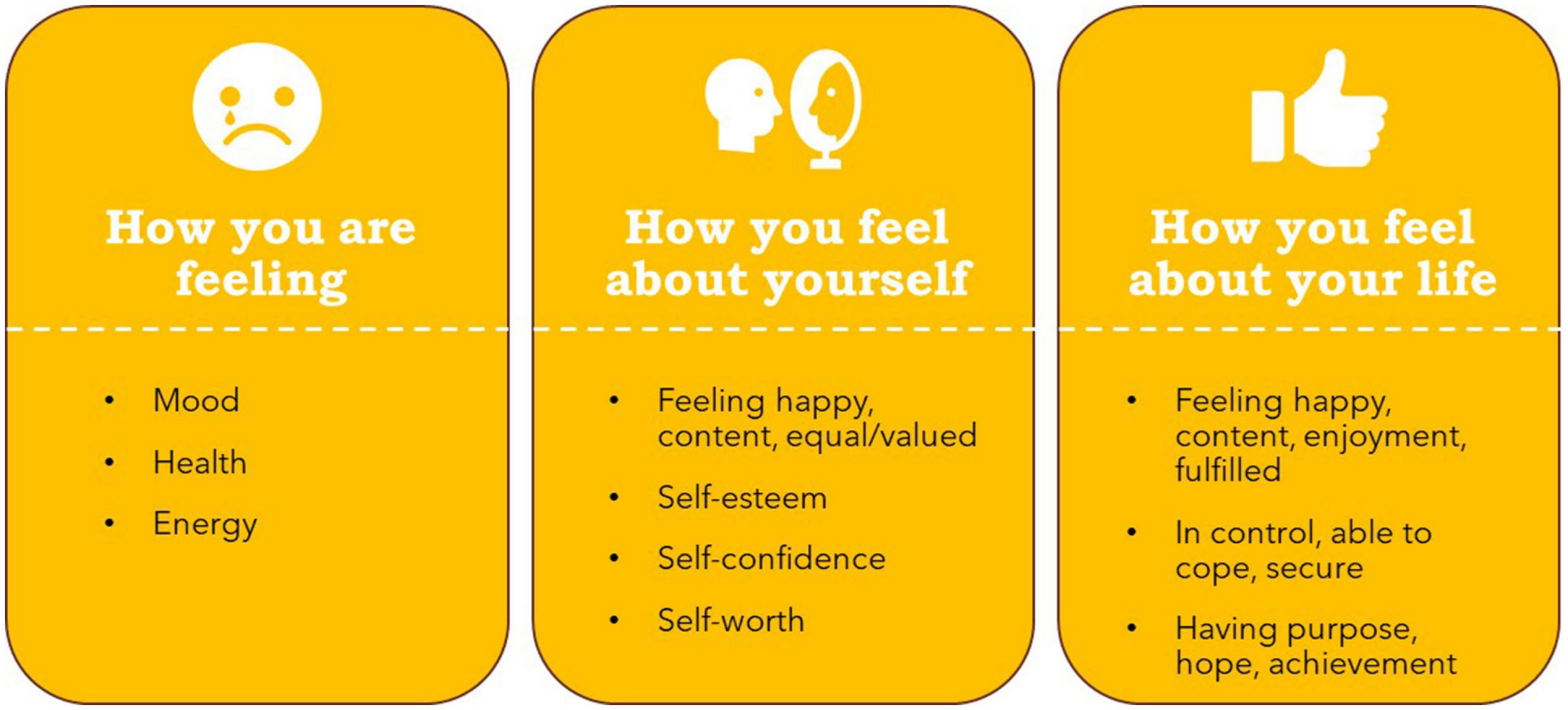
Components of well-being.

Several participants across different groups equated well-being with contentment, or its alternative happiness or feeling good. The core of well-being may, therefore, consist of an overall feeling of contentment, which in itself consists of a positive evaluation of how one is feeling, how one is feeling about or within oneself, and how one is feeling about their life (“*And I think that [achieving your own goals] has a huge effect on how you feel about yourself and how the day and your life is going, quite frankly*”—**WB09, FG01**). The components of well-being identified by participants can be grouped into these three categories (Figure 1). How a person feels reflects an evaluation of mental and physical health, mood, and energy levels. However, mood may also be a factor or form part of the protective buffer. Good well-being, therefore, reflects feeling healthy in body and mind, an absence of negative and presence of positive affect, and having the energy and motivation to do what one wants to do. For many participants, their definition of well-being included an evaluation of how they felt about themselves (“*Well-being is somewhere around how we cope and how we cope is how we feel about ourselves on a longer basis*”—**WB09, FG01**). In this category, well-being was related to self-esteem, self-confidence, and self-worth. Good well-being then reflected the extent to which people felt good, happy, content, comfortable, and confident within and about themselves and the extent to which they valued and loved themselves. Components relating to an evaluation of one’s life include the extent to which people feel good about their lives. This includes an evaluation of one’s immediate life and life in general. Good well-being, therefore, reflects the extent to which people felt content, happy, good, and satisfied with their lives; the extent to which they felt fulfilled and secure; the extent to which they felt in control and able to cope with difficulties; and the extent to which their lives contained a sense of enjoyment, hope, purpose, and achievement.

**Figure 2:**
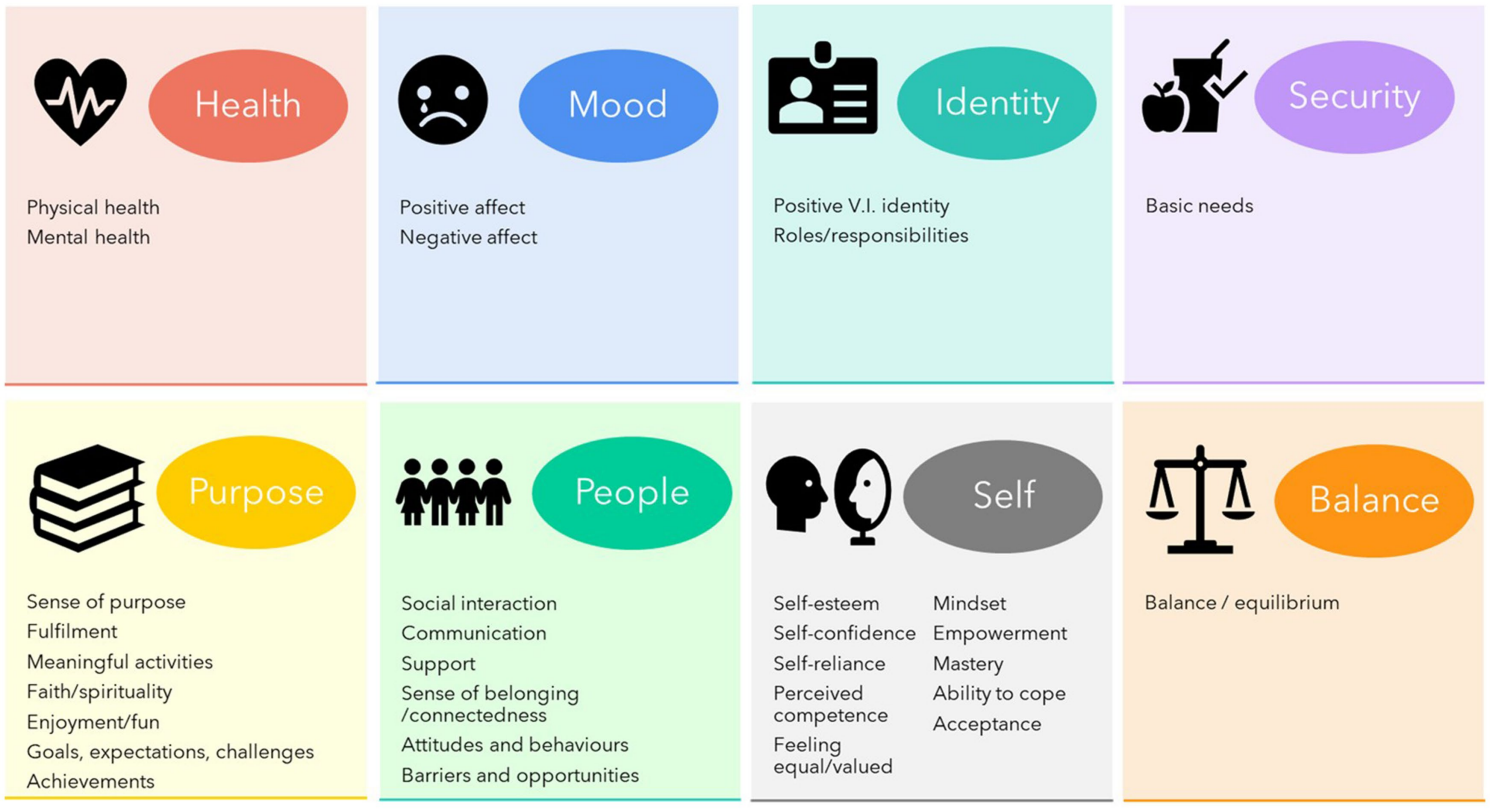
Factors and associated items which impact on well-being.

Participants further identified various factors relating to other people, health, mood, security, the self, identity, having purpose, and balance/equilibrium. These factors can, at various times, but do not have to, impact well-being (Figure 2).

**Figure 3:**
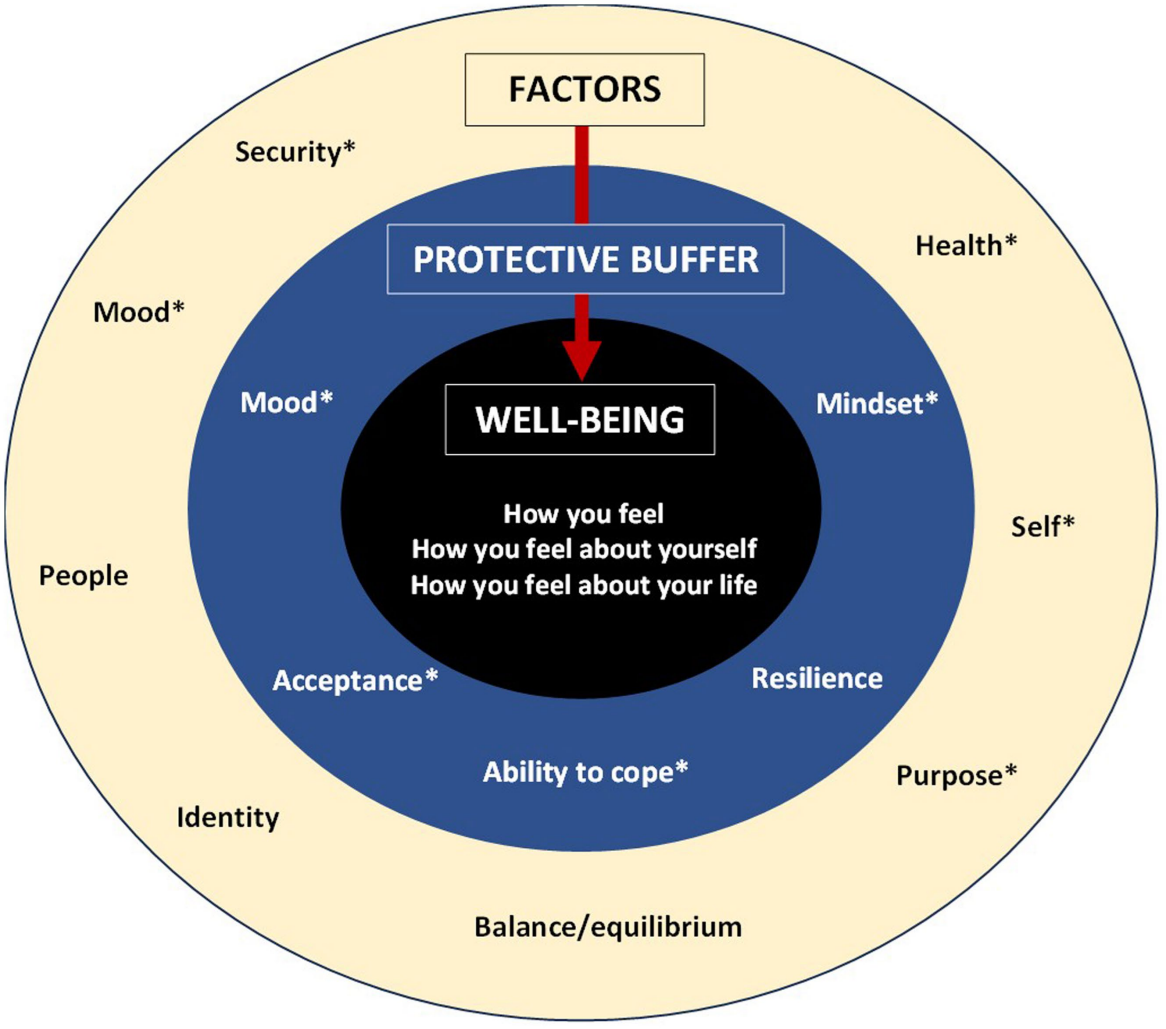
Model of well-being consisting of factors, a protective buffer, and the core of well-being (the symbol * denotes items with unclear roles).

Moreover, their impact may be mitigated by the protective buffer, which may consist of a person’s mood, ability to cope, resilience, and mindset (Figure 3).

While the core of well-being is proposed to be universal but changeable, the factors impacting well-being are proposed to be individual and changeable across time. Although the core is proposed to be universal, there is variation in how each component is experienced by individuals requiring baseline measurements.

As such, the core of well-being in this proposed model contains elements of hedonic models, such as Diener and colleagues’ SWB, which consists of affect and life satisfaction (Diener, 1984; Diener and Suh, 1997), while some of the factors reflect elements included in eudaimonic models of well-being. For instance, the self-related items self-reliance, mastery, acceptance, and perceived competence, along with items associated with other people, purpose in life, and achievements, are reflective of autonomy, mastery, positive relationships with others, purpose in life, and self-acceptance in the PWB (Ryff, 1989), autonomy, competence, and relatedness in the SDT (Ryan and Deci, 2001), and engagement, relationships, meaning, and accomplishments in the PERMA model (Seligman, 2012). The latter also includes a hedonic element of positive emotions. The individual items identified by participants are, therefore, not new, but the proposed structure of the model is. Rather than being components of well-being, eudaimonic elements of well-being are proposed to be factors or items that mitigate the impact of factors on well-being in the current model.

While the proposed model may be applicable across populations, differences may emerge when applying the model to the assessment and support of well-being. As discussed, V.I. can impact how individuals feel about themselves and their lives, as well as on a range of other factors. These would need to be taken into consideration when identifying or designing a measure to assess well-being in adults with V.I.

### 4.2 Limitations and next steps

A main limitation is that the study did not include a comparison of people with V.I. and the general population. While this was outside the scope of this study, it should be a focus of future research. Furthermore, the findings are based on a small convenience sample of participants recruited through V.I. charities. The practitioner group was remarkably homogenous. Future research could use the Delphi method to confirm the findings and determine the exact role of individual components in a larger, more representative sample. Moreover, a Delphi approach will enable a comparison between the definitions of well-being provided by practitioners and adults with V.I., as well as a quantitative measure of the importance of individual items. Moreover, it would allow for the collection of more sample characteristics, including physical and cognitive functioning, which were not collected in the current qualitative study.

Coding is an intrinsically subjective process. It is possible that some items and their roles have not been coded as intended by the participants. Moreover, the items could have been labeled and grouped in other ways. In the first instance, more research is required to confirm and refine the items identified in these FGs and determine the exact role of items such as mood, health, and balance/equilibrium. Once the items have been confirmed, the structure of the proposed model, consisting of a core of well-being, factors, and the protective buffer, will need to be tested and confirmed with people with lived experience of V.I. before work on identifying or designing a potential tool to assess well-being can begin. For instance, mediation and moderation analysis could be used to test if items in the protective buffer are mediators, which account for the relationship between a predictor and an outcome variable, or moderators, which impact “the direction and/or strength of the relation” between a predictor and an outcome variable (Baron and Kenny, 1986; Nima et al., 2013). A further layer to this model may consist of interventions which can improve individual factors and, thus, well-being. These may include the activities listed by participants. For instance, journaling may aid self-awareness and thus improve acceptance or self-esteem. Similarly, going for a walk may improve physical and mental health. However, these were highly individual, as one participant noted, the ‘one-size-fits-all approach’ may not work.

## 5 Conclusion

Having a shared understanding of well-being, which reflects the experiences of people with lived experience and is flexible enough to allow for individual differences is vital to enabling cooperation between support organizations within the sector. Taking a bottom-up approach, this article proposes a preliminary model of well-being in the context of V.I. based on items identified by people with lived experience of V.I. and practitioners working with them. The model and the items of which it comprises will need to be tested in future research.

## Data Availability

The datasets presented in this article are not readily available because participants were not asked for consent to share data with anyone outside the research team. Requests to access the datasets should be directed to renata.gomes@bravovictor.org.
