# Supplementary material for: What do adults with visual impairment mean by well-being? Identifying the building blocks of well-being in the context of visual impairment
Source: Front Psychol. 2024 Aug 22;15:1395636. doi: 10.3389/fpsyg.2024.1395636 (PMC11375082; doi:10.3389/fpsyg.2024.1395636)
Supplement: Supplementary file 1 [file Table_1.DOCX]

**Topic Guide – Adults with V.I.**

*Agree ground rules with group*

Normally we would start with an icebreaker, but we have quite a lot of ground to cover so we are going to go straight in it and as our icebreaker we would like to go around the room and ask you:

1. What do you mean when you talk about well-being? *Remember there are no right or wrong answers at all.*
   **Probes:**
   1. Do you think X is well-being or is it a factor that impacts on your well-being? [Give explanation of what is a factor and what is component.]
   2. How do you know if your well-being is good or poor?
   3. How do you talk about well-being? Do you say it’s good or bad or poor, high or low,…
   4. How do you assess your well-being?
   5. Do you think well-being is different for people with visual impairment than for those without or is it the same? Could you describe how (if yes)?
   6. Do you feel that your visual impairment impacts on your well-being? Could you describe how (if yes)?
2. What are the factors that you feel impact on your own well-being?

**Probes:**

- 1. For example, some people say that factors such as finances, relationships, family, work, health impact on their well-being
  2. Which of these are most important?

1. Considering all that we have talked about today, how would you describe what well-being means to an alien who has just arrived on this planet?

**Topic Guide – Practitioners**

*Agree ground rules with group*

Normally we would start with an icebreaker, but we have quite a lot of ground to cover so we are going to go straight in it and as our icebreaker we would like to go around the room and ask you:

1. What do you mean when you talk about well-being? *Remember there are no right or wrong answers at all.*
   **Probes:**
   1. Do you think that well-being is different or the same for people with visual impairment?
2. We are also interested in the factors which impact on well-being. First of all, which factors would you say have a negative impact on well-being in people with visual impairment?
3. And which factors would you say have a positive impact on well-being in people with visual impairment?

**Probes:**

- 1. Which of these factors are most important in your experience?
  2. To what extent are these factors the same for everyone?
  3. To what extent are these factors stable?

1. How have you assessed well-being in people with visual impairment?

**Probes:**

- 1. Which tools have you used to assess well-being
  2. What has your experience been of using different tools to assess well-being? Good and bad
  3. How do you know if well-being has improved or deteriorated?

1. Considering everything we have talked about today, how would you describe what well-being means to an alien who has just arrived on this planet?
